# Diaqua­bis{[1-hydr­oxy-2-(1*H*-imidazol-3-ium-1-yl)ethane-1,1-di­yl]bis­(hydrogen phospho­nato)}manganese(II)

**DOI:** 10.1107/S160053680905065X

**Published:** 2009-11-28

**Authors:** Zai-Chao Zhang, Rong-Qing Li, Yu Zhang

**Affiliations:** a Jiangsu Key Laboratory for the Chemistry of Low-dimensional Materials, Department of Chemistry, Huaiyin Teachers College, 111 West Changjiang Road, Huaian 223300, Jiangsu, People’s Republic of China

## Abstract

In the title compound, [Mn(C_5_H_9_N_2_O_7_P_2_)_2_(H_2_O)_2_], the Mn^II^ atom (site symmetry 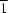) is coordinated by four phos­pho­n­ate O atoms from a pair of partially deprotonated 1-hydr­oxy-2-(imidazol-3-yl)ethane-1,1-bis­phophonic acid ligands (imhedpH_3_^−^) and two water mol­ecules, resulting in a slightly distorted *trans*-MnO_6_ octa­hedral geometry for the metal ion. In the ligands, the imidazole units are protonated and two of the hydr­oxy O atoms of the phospho­nate groups are deprotonated and chelate the Mn^II^, thus forming the neutral mol­ecule of the title compound. The two protonated O atoms within the phospho­nate groups of one imhedpH_3_^−^ ligand act as hydrogen-bond acceptors for a bifurcated hydrogen bond originating from the coordinated water mol­ecule. The phospho­nate units of neigboring mol­ecules are connected with their equivalents in neighboring mol­ecules *via* two types of inversion-symmetric hydrogen-bonding arrangements with four and two strong O—H⋯O hydrogen bonds, respectively. The two inter­actions connect mol­ecules into infinite chains along [111] and [110], in combination forming a tightly hydrogen-bonded three-dimensional supra­molecular network. This network is further stabilized by additional hydrogen bonds between the protonated imidazole units and one of the coordinated P—O O atoms and by additional O—H⋯O hydrogen bonds between the water mol­ecules and the P=O O atoms of neigboring mol­ecules.

## Related literature

For a review of the structures and applications of lanthanide phospho­nates, see: Mao (2007 ▶). For other complexes based on the imhedpH_4_ ligand, see: Cao *et al.* (2007 ▶, 2008 ▶). For the structures and properties of some metal organophospho­nates, see: Rao *et al.* (2004 ▶); Yang *et al.* (2009 ▶).
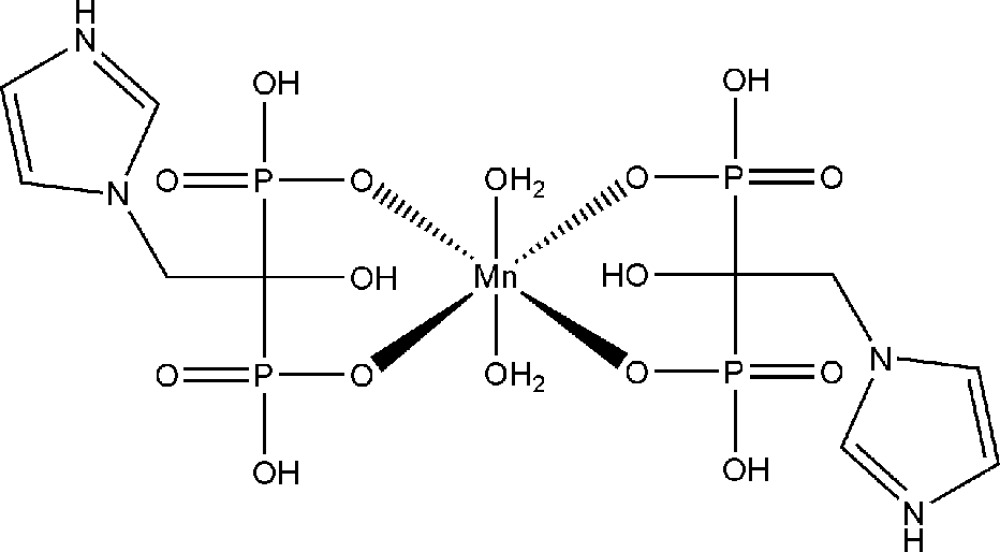


## Experimental

### 

#### Crystal data

[Mn(C_5_H_9_N_2_O_7_P_2_)_2_(H_2_O)_2_]
*M* *_r_* = 633.14
Triclinic, 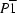

*a* = 7.4408 (17) Å
*b* = 8.566 (2) Å
*c* = 9.680 (2) Å
α = 105.366 (4)°
β = 110.865 (4)°
γ = 97.461 (4)°
*V* = 538.4 (2) Å^3^
*Z* = 1
Mo *K*α radiation
μ = 1.00 mm^−1^
*T* = 153 K
0.25 × 0.20 × 0.20 mm

#### Data collection

Bruker SMART APEXII diffractometer
Absorption correction: multi-scan (*SADABS*; Bruker, 2000 ▶) *T*_min_ = 0.788, *T*_max_ = 0.825
2637 measured reflections
1829 independent reflections
1543 reflections with *I* > 2σ(*I*)
*R*_int_ = 0.029

#### Refinement

*R*[*F* ^2^ > 2σ(*F* ^2^)] = 0.052
*wR*(*F* ^2^) = 0.135
*S* = 0.99
1829 reflections
175 parameters
H atoms treated by a mixture of independent and constrained refinement
Δρ_max_ = 0.49 e Å^−3^
Δρ_min_ = −0.46 e Å^−3^

### 

Data collection: *APEX2* (Bruker, 2004 ▶); cell refinement: *SAINT* (Bruker, 2004 ▶); data reduction: *SAINT*; program(s) used to solve structure: *SHELXS97* (Sheldrick, 2008 ▶); program(s) used to refine structure: *SHELXL97* (Sheldrick, 2008 ▶); molecular graphics: *ORTEP-3 for Windows* (Farrugia, 1997 ▶); software used to prepare material for publication: *SHELXL97* and *PLATON* (Spek, 2009 ▶).

## Supplementary Material

Crystal structure: contains datablocks global, I. DOI: 10.1107/S160053680905065X/zl2255sup1.cif

Structure factors: contains datablocks I. DOI: 10.1107/S160053680905065X/zl2255Isup2.hkl

Additional supplementary materials:  crystallographic information; 3D view; checkCIF report

## Figures and Tables

**Table 1:** Hydrogen-bond geometry (Å, °)

*D*—H⋯*A*	*D*—H	H⋯*A*	*D*⋯*A*	*D*—H⋯*A*
N2—H2*A*⋯O4^i^	0.88	1.85	2.718 (5)	167
O7—H7*A*⋯O5^ii^	0.85 (5)	2.07 (5)	2.889 (4)	162 (5)
O6—H6*A*⋯O5^ii^	0.84 (5)	1.82 (5)	2.653 (4)	167 (5)
O3—H3*A*⋯O2^iii^	0.85 (5)	1.74 (5)	2.573 (4)	167 (5)
O8—H8*A*⋯O2^iv^	0.86 (6)	1.88 (6)	2.707 (5)	162 (5)
O8—H8*B*⋯O3	0.85 (6)	2.32 (6)	3.042 (5)	143 (5)
O8—H8*B*⋯O6	0.85 (6)	2.59 (6)	3.124 (5)	122 (5)

Symmetry codes: (i) 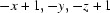; (ii) 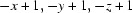; (iii) 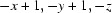; (iv) 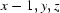.

**Fig. 1.**
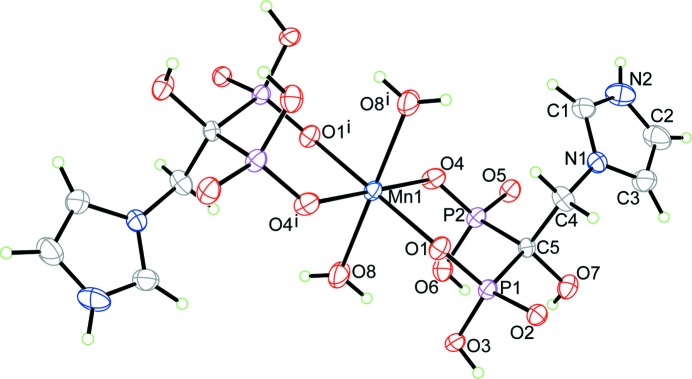
The molecular view of the title compound with atomic labeling scheme (50% probability, symmetry code: i, –x, –y, –z).

**Fig. 2.**
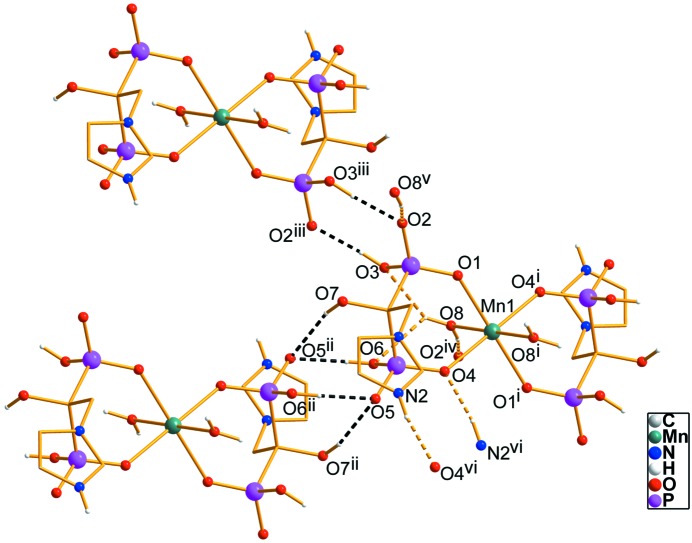
A view of the hydrogen bonding interactions in the structure of the title compound based on its asymmetric unit. Hydrogen bonding interactions within the centrosymmetric units (see comment) are represented by black dashed lines, other as dashed yellow lines (symmetry codes: i, –x, –y, –z; ii, 1–x, 1–y, 1–z; iii, 1–x, 1–y, –z; iv, –1+x, y, z; v, 1+x, y, z; vi, 1–x, –y, 1–z).

**Fig. 3.**
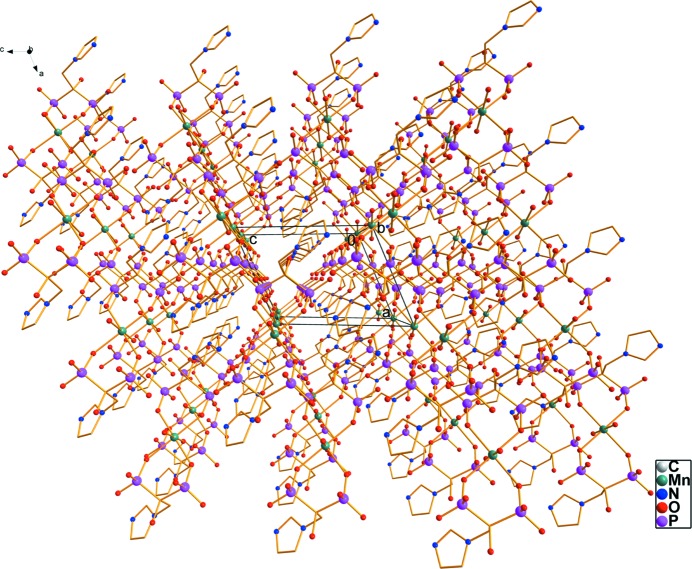
Packing diagram of the title compound.

## supplementary crystallographic information

### Comment

Organophosphonic acids have attracted much attention in the field of organic-inorganic hybrid materials because of their flexible coordination modes and strong coordination ability (Mao 2007; Rao *et. al.* 2008; Yang *et al.* 2009). Compared to other organophosphonic acid derivatives, there are only few complexes of metal-organic compounds with 1-hydroxy-2-(imidazol-3-yl)ethane-1,1-bisphophonic acid (imhedpH_4_) and its anions as the ligand (Cao *et al.* 2007; Cao *et al.* 2008). Herein, we thus report the synthesis and structure of the title compound, a manganese complex of the monoanion of the aformentioned acid.

The title compound crystallizes in the triclinic system with the space group *P*1. The Mn^II^ ion adopts a slightly distorted octahedral geometry and is located on a crystallographic inversion center. Two pairs of phosphonate oxygen atoms (O1, O4, O1^i^, O4^i^; symmetry code: i, –x, –y, –z) from two equivalent imhedpH_3_^-^ ligands define the equatorial plane, while two equivalent water molecules (O8 and O8^i^) occupy the axial sites. The Mn-O bond lengths are in the range of 2.118 (3)-2.218 (3) Å. In the equatorial plane, the O—Mn—O bond angles are 88.35 (11)-91.65 (11)°. The two imhedpH_3_^-^ ligands act as bidentate chelating ligands towards the Mn cation via the two deprotonated phosphonate oxygen atoms O1 and O4, while two of the remaining four phosphonate oxygens are protonated [P1—O3 = 1.565 (3) Å and P2—O6 = 1.572 (3) Å]. These two protonated O atoms O3 and O6 act as a hydrogen bond acceptors for a bifurcated H bond from the coordinated water molecule (O8, see Table 1 for numerical values).

The phosphonate units are connected with their equivalents in neighboring molecules via two types of inversion symmetric hydrogen bonding arrangements (Fig. 2). Phosphonate units of P atom P1 are connecting neighboring molecules along the diagonal of the unit cell by means of four strong O—H···O hydrogen bonds between the P—O—H (O6), C—O—H (O7) and P═O units (O5) of the imhedpH_3_^-^ ligands (Table 1). Dimeric P—O—H···O═P connections are formed between the phosphonate units of P2 in neighboring molecules involving O2 and O3. This second interaction connects molecules along the {1 1 0} direction, and both of these strong hydrogen bonding interactions lead to the formation of infinite chains, and in combination to a tightly hydrogen bonded three-dimensional supramolecular network (Fig. 3). This network is further stabilized by additional hydrogen bonds between the protonated imidazole units towards one of the coordinated P-O oxygen atoms (O4) and by additional O—H···O hydrogen bonds between the water molecule and P═O oxygen atoms (O2) of neigboring molecules (Table 1).

### Experimental

The title compound was prepared by the hydrothermal reaction of a mixture of MnSO_4_ (0.1 mmol), imhedpH_4_ (0.1 mmol) and H_2_O (8.0 mL) in a Teflon-lined stainless steel autoclave (25 ml), which was heated to 413 K for 48 h. Block-shaped light-pink crystals were collected (yield: 48%, based on imhedpH_4_). Anal. calcd for C_10_H_22_MnN_4_O_16_P_4_ (633.13): C 18.97, H 3.50, N 8.85%; found: C 19.06, H 3.71, N 8.90%.

### Refinement

All non-hydrogen atoms were found in Fourier maps and were refined anisotropically. Hydrogen atoms attached to C and N atoms were positioned geometrically with bond distances of 0.95 or 0.99 Å for C—H and 0.88 Å for N—H. Water and hydroxy H atoms were located in a difference Fourier map and refined with distance restraints on all O—H bond lengths with distances of 0.85 (1) Å, and the H···H distance within the water molecule was restrained to 1.55 (2) Å. The isotropic displacement parameters of H atoms are related to the non-H atom to which they are bonded, *viz*. *U*_iso_(H) = 1.2 *U*_eq_(parent).

### Crystal data

**Table:**

[Mn(C_5_H_9_N_2_O_7_P_2_)_2_(H_2_O)_2_]	*Z* = 1
*M**_r_* = 633.14	*F*(000) = 323
Triclinic, *P*1	*D*_x_ = 1.953 Mg m^−^^3^
Hall symbol: -P 1	Mo *K*α radiation, λ = 0.71073 Å
*a* = 7.4408 (17) Å	Cell parameters from 584 reflections
*b* = 8.566 (2) Å	θ = 2.4–24.6°
*c* = 9.680 (2) Å	µ = 1.00 mm^−^^1^
α = 105.366 (4)°	*T* = 153 K
β = 110.865 (4)°	Block, light pink
γ = 97.461 (4)°	0.25 × 0.20 × 0.20 mm
*V* = 538.4 (2) Å^3^	

### Data collection

**Table:**

Bruker SMART APEXII diffractometer	1829 independent reflections
Radiation source: fine-focus sealed tube	1543 reflections with *I* > 2σ(*I*)
graphite	*R*_int_ = 0.029
ω scans	θ_max_ = 25.0°, θ_min_ = 2.4°
Absorption correction: multi-scan (*SADABS*; Bruker, 2000)	*h* = −8→4
*T*_min_ = 0.788, *T*_max_ = 0.825	*k* = −10→10
2637 measured reflections	*l* = −9→11

### Refinement

**Table:**

Refinement on *F*^2^	Primary atom site location: structure-invariant direct methods
Least-squares matrix: full	Secondary atom site location: difference Fourier map
*R*[*F*^2^ > 2σ(*F*^2^)] = 0.052	Hydrogen site location: inferred from neighbouring sites
*wR*(*F*^2^) = 0.135	H atoms treated by a mixture of independent and constrained refinement
*S* = 0.99	*w* = 1/[σ^2^(*F*_o_^2^) + (0.0806*P*)^2^ + 0.0364*P*] where *P* = (*F*_o_^2^ + 2*F*_c_^2^)/3
1829 reflections	(Δ/σ)_max_ < 0.001
175 parameters	Δρ_max_ = 0.49 e Å^−^^3^
0 restraints	Δρ_min_ = −0.46 e Å^−^^3^

### Special details

**Table:**

Geometry. All esds (except the esd in the dihedral angle between two l.s. planes) are estimated using the full covariance matrix. The cell esds are taken into account individually in the estimation of esds in distances, angles and torsion angles; correlations between esds in cell parameters are only used when they are defined by crystal symmetry. An approximate (isotropic) treatment of cell esds is used for estimating esds involving l.s. planes.
Refinement. Refinement of F^2^ against ALL reflections. The weighted R-factor wR and goodness of fit S are based on F^2^, conventional R-factors R are based on F, with F set to zero for negative F^2^. The threshold expression of F^2^ > 2sigma(F^2^) is used only for calculating R-factors(gt) etc. and is not relevant to the choice of reflections for refinement. R-factors based on F^2^ are statistically about twice as large as those based on F, and R- factors based on ALL data will be even larger.

### Fractional atomic coordinates and isotropic or equivalent isotropic displacement parameters (Å^2^)

**Table:**

	*x*	*y*	*z*	*U*_iso_*/*U*_eq_	
C1	0.7059 (7)	0.0407 (6)	0.4433 (6)	0.0313 (11)	
H1	0.5957	−0.0541	0.3933	0.038*	
C2	0.9765 (8)	0.2242 (6)	0.6170 (6)	0.0411 (13)	
H2	1.0898	0.2812	0.7125	0.049*	
C3	0.9192 (7)	0.2695 (6)	0.4880 (5)	0.0330 (12)	
H3	0.9850	0.3632	0.4743	0.040*	
C4	0.6265 (7)	0.1604 (6)	0.2269 (5)	0.0263 (10)	
H4A	0.5289	0.0511	0.1646	0.032*	
H4B	0.7133	0.1768	0.1719	0.032*	
C5	0.5147 (6)	0.2977 (5)	0.2313 (5)	0.0186 (9)	
Mn1	0.0000	0.0000	0.0000	0.0188 (3)	
N1	0.7483 (5)	0.1543 (4)	0.3809 (4)	0.0231 (8)	
N2	0.8436 (7)	0.0832 (5)	0.5856 (5)	0.0393 (11)	
H2A	0.8485	0.0279	0.6511	0.047*	
O1	0.2208 (4)	0.1161 (3)	−0.0561 (3)	0.0218 (7)	
O2	0.5292 (4)	0.2914 (3)	−0.0465 (3)	0.0212 (7)	
O3	0.2772 (4)	0.4247 (4)	0.0239 (4)	0.0236 (7)	
H3A	0.355 (7)	0.511 (6)	0.031 (5)	0.028*	
O4	0.1983 (4)	0.1153 (4)	0.2449 (3)	0.0256 (7)	
O5	0.4729 (4)	0.3069 (4)	0.5030 (3)	0.0258 (7)	
O6	0.2356 (5)	0.4203 (4)	0.3166 (3)	0.0258 (7)	
H6A	0.316 (7)	0.515 (6)	0.369 (6)	0.031*	
O7	0.6604 (5)	0.4552 (4)	0.3074 (4)	0.0274 (8)	
H7A	0.616 (7)	0.534 (6)	0.347 (6)	0.033*	
O8	−0.1140 (6)	0.2247 (5)	−0.0097 (5)	0.0421 (10)	
H8A	−0.232 (9)	0.226 (7)	−0.018 (7)	0.051*	
H8B	−0.021 (9)	0.314 (7)	0.028 (7)	0.051*	
P1	0.37848 (16)	0.27554 (13)	0.02248 (13)	0.0187 (3)	
P2	0.34950 (16)	0.27977 (13)	0.33400 (12)	0.0203 (3)	

### Atomic displacement parameters (Å^2^)

**Table:**

	*U*^11^	*U*^22^	*U*^33^	*U*^12^	*U*^13^	*U*^23^
C1	0.032 (3)	0.032 (3)	0.038 (3)	0.011 (2)	0.017 (2)	0.020 (2)
C2	0.041 (3)	0.036 (3)	0.030 (3)	0.008 (3)	−0.003 (2)	0.011 (2)
C3	0.031 (3)	0.029 (3)	0.030 (3)	0.001 (2)	0.004 (2)	0.011 (2)
C4	0.027 (3)	0.030 (3)	0.020 (2)	0.013 (2)	0.008 (2)	0.006 (2)
C5	0.015 (2)	0.020 (2)	0.016 (2)	0.0023 (18)	0.0028 (18)	0.0052 (17)
Mn1	0.0177 (5)	0.0178 (5)	0.0178 (5)	0.0005 (4)	0.0058 (4)	0.0047 (4)
N1	0.021 (2)	0.025 (2)	0.022 (2)	0.0057 (16)	0.0065 (17)	0.0092 (16)
N2	0.052 (3)	0.045 (3)	0.031 (2)	0.016 (2)	0.017 (2)	0.026 (2)
O1	0.0219 (17)	0.0240 (17)	0.0183 (16)	0.0013 (13)	0.0098 (13)	0.0051 (13)
O2	0.0197 (16)	0.0242 (16)	0.0232 (16)	0.0056 (13)	0.0111 (13)	0.0099 (13)
O3	0.0211 (17)	0.0222 (17)	0.0342 (18)	0.0055 (13)	0.0143 (15)	0.0155 (14)
O4	0.0316 (18)	0.0241 (17)	0.0153 (16)	−0.0009 (14)	0.0062 (14)	0.0061 (13)
O5	0.0251 (17)	0.0299 (18)	0.0168 (16)	0.0001 (14)	0.0063 (14)	0.0057 (13)
O6	0.0223 (18)	0.0273 (18)	0.0225 (17)	0.0041 (14)	0.0080 (14)	0.0027 (14)
O7	0.0222 (17)	0.0244 (18)	0.0294 (18)	0.0004 (14)	0.0095 (15)	0.0035 (14)
O8	0.023 (2)	0.026 (2)	0.077 (3)	0.0073 (16)	0.021 (2)	0.0163 (19)
P1	0.0175 (6)	0.0197 (6)	0.0188 (6)	0.0028 (5)	0.0078 (5)	0.0065 (5)
P2	0.0211 (6)	0.0205 (6)	0.0162 (6)	0.0009 (5)	0.0073 (5)	0.0038 (5)

### Geometric parameters (Å, °)

**Table:**

C1—N2	1.309 (6)	Mn1—O4	2.159 (3)
C1—N1	1.334 (6)	Mn1—O4^i^	2.159 (3)
C1—H1	0.9500	Mn1—O8^i^	2.213 (4)
C2—N2	1.345 (6)	Mn1—O8	2.213 (4)
C2—C3	1.347 (6)	N2—H2A	0.8800
C2—H2	0.9500	O1—P1	1.489 (3)
C3—N1	1.363 (6)	O2—P1	1.505 (3)
C3—H3	0.9500	O3—P1	1.565 (3)
C4—N1	1.462 (5)	O3—H3A	0.85 (5)
C4—C5	1.526 (6)	O4—P2	1.499 (3)
C4—H4A	0.9900	O5—P2	1.499 (3)
C4—H4B	0.9900	O6—P2	1.571 (3)
C5—O7	1.437 (5)	O6—H6A	0.84 (5)
C5—P2	1.850 (4)	O7—H7A	0.85 (5)
C5—P1	1.854 (4)	O8—H8A	0.86 (6)
Mn1—O1	2.116 (3)	O8—H8B	0.85 (6)
Mn1—O1^i^	2.116 (3)		
			
N2—C1—N1	107.7 (4)	O1—Mn1—O8	84.33 (13)
N2—C1—H1	126.2	O1^i^—Mn1—O8	95.67 (13)
N1—C1—H1	126.2	O4—Mn1—O8	93.01 (13)
N2—C2—C3	107.3 (4)	O4^i^—Mn1—O8	86.99 (13)
N2—C2—H2	126.3	O8^i^—Mn1—O8	180.0
C3—C2—H2	126.3	C1—N1—C3	108.7 (4)
C2—C3—N1	106.5 (4)	C1—N1—C4	125.9 (4)
C2—C3—H3	126.8	C3—N1—C4	125.4 (4)
N1—C3—H3	126.8	C1—N2—C2	109.9 (4)
N1—C4—C5	114.6 (3)	C1—N2—H2A	125.1
N1—C4—H4A	108.6	C2—N2—H2A	125.1
C5—C4—H4A	108.6	P1—O1—Mn1	134.48 (17)
N1—C4—H4B	108.6	P1—O3—H3A	111 (3)
C5—C4—H4B	108.6	P2—O4—Mn1	132.06 (17)
H4A—C4—H4B	107.6	P2—O6—H6A	110 (3)
O7—C5—C4	107.3 (3)	C5—O7—H7A	113 (3)
O7—C5—P2	111.1 (3)	Mn1—O8—H8A	121 (4)
C4—C5—P2	112.1 (3)	Mn1—O8—H8B	113 (4)
O7—C5—P1	108.2 (3)	H8A—O8—H8B	123 (5)
C4—C5—P1	104.7 (3)	O1—P1—O2	115.48 (17)
P2—C5—P1	113.0 (2)	O1—P1—O3	108.67 (17)
O1—Mn1—O1^i^	180.00 (17)	O2—P1—O3	110.18 (16)
O1—Mn1—O4	88.32 (11)	O1—P1—C5	108.75 (17)
O1^i^—Mn1—O4	91.68 (11)	O2—P1—C5	107.50 (18)
O1—Mn1—O4^i^	91.68 (11)	O3—P1—C5	105.82 (18)
O1^i^—Mn1—O4^i^	88.32 (11)	O5—P2—O4	115.53 (17)
O4—Mn1—O4^i^	180.00 (17)	O5—P2—O6	111.28 (17)
O1—Mn1—O8^i^	95.67 (13)	O4—P2—O6	107.09 (18)
O1^i^—Mn1—O8^i^	84.33 (13)	O5—P2—C5	109.22 (18)
O4—Mn1—O8^i^	86.99 (13)	O4—P2—C5	107.87 (18)
O4^i^—Mn1—O8^i^	93.01 (13)	O6—P2—C5	105.31 (18)

Symmetry codes: (i) −*x*, −*y*, −*z*.

### Hydrogen-bond geometry (Å, °)

**Table:**

*D*—H···*A*	*D*—H	H···*A*	*D*···*A*	*D*—H···*A*
N2—H2A···O4^ii^	0.88	1.85	2.718 (5)	167
O7—H7A···O5^iii^	0.85 (5)	2.07 (5)	2.889 (4)	162 (5)
O6—H6A···O5^iii^	0.84 (5)	1.82 (5)	2.653 (4)	167 (5)
O3—H3A···O2^iv^	0.85 (5)	1.74 (5)	2.573 (4)	167 (5)
O8—H8A···O2^v^	0.86 (6)	1.88 (6)	2.707 (5)	162 (5)
O8—H8B···O3	0.85 (6)	2.32 (6)	3.042 (5)	143 (5)
O8—H8B···O6	0.85 (6)	2.59 (6)	3.124 (5)	122 (5)

Symmetry codes: (ii) −*x*+1, −*y*, −*z*+1; (iii) −*x*+1, −*y*+1, −*z*+1; (iv) −*x*+1, −*y*+1, −*z*; (v) *x*−1, *y*, *z*.
